# Survivorship and Etiologies of Failure in Single-stage Revision Arthroplasty for Periprosthetic Joint Infection: A Meta-analysis

**DOI:** 10.5435/JAAOSGlobal-D-22-00218

**Published:** 2023-05-11

**Authors:** Kranti V. Peddada, Brandon M. Welcome, Mitchell C. Parker, Connor M. Delman, Christopher T. Holland, Mauro Giordani, John P. Meehan, Zachary C. Lum

**Affiliations:** From the Department of Orthopaedic Surgery, Davis Medical Center, University of California (Dr. Peddada, Dr. Delman, Dr. Holland, Dr. Giordani, Dr. Meehan, and Dr. Lum), and the Reno School of Medicine, University of Nevada (Mr. Welcome, and Mr. Parker).

## Abstract

Single-stage revision arthroplasty for periprosthetic joint infection (PJI) may yield comparable infection-free survivorship with two-stage revision arthroplasty. It is unclear if the most common mode of failure of single-stage revision arthroplasty is infection or aseptic loosening. In this meta-analysis, we sought to (1) determine survivorship and (2) compare rates of different etiologies of failure of single-stage revision total hip arthroplasty (THA) and total knee arthroplasty (TKA).

**Methods:**

Preferred Reporting Items for Systematic Review and Meta-analyses guidelines search was done using search terms for “single stage revision,” “exchange arthroplasty,” “periprosthetic infection,” “PJI,” and “single stage.” Patient demographics such as age, body mass index, and mean follow-up time were recorded. Overall survivorship and rates of revision surgery were aggregated using a random-effects model. Comparison of septic and aseptic loosening rates was done by risk difference and associated 95% confidence interval (CI) calculation.

**Results:**

Twenty-four studies were identified with 2,062 and 147 single-stage revision THA and TKA procedures performed between 1984 and 2019, respectively. The weighted mean follow-up and age were 69.8 months and 66.3 years, respectively, with 55% men overall. The all-cause revision surgery rate was 11.1% and 11.8% for THA and TKA, respectively. The revision surgery rate secondary to infection and aseptic loosening and associated 95% CI for the risk difference for THA and TKA was 5.5% and 3.3% (−1.7% to 5.0%), and 3% and 8.8% (−11.4% to 2.3%), respectively. Revision surgeries due to instability and fracture combined and mortality rate were both less than 3%.

**Discussion:**

Single-stage revision THA and TKA for PJI demonstrated overall high rates of survivorship, low mortality, and revision surgeries secondary to infection and aseptic loosening to be equivalent. Aseptic loosening after single-stage revision TKA might be higher than in primary TKA. As implant survivorship from infection improves in PJI, surgeons should be aware of aseptic loosening as an equally common mode of failure.

One of the most common etiologies of total knee arthroplasty (TKA) and total hip arthroplasty (THA) revision is infection. The cumulative incidence of prosthetic joint infection (PJI) is approximately 5% at 2 years for TKA and 1.5% at 15 years for THA.^1,2^ Single-stage revision has emerged as a popular surgical management strategy for chronic PJI. Recent studies have demonstrated equivalent infection-free success compared to two-staged revision, while incurring less morbidity and mortality, fewer hospitalizations, shorter antibiotic duration, and lower health-care costs.^3^ Aseptic loosening is an important cause of revision surgery in single-stage revision with associated failure rates as high as 41%.^4,5,6,7^ The purpose of this study was to determine the survivorship and compare rates of different etiologies of failure of single-stage revision THA and TKA.

## Methods

This meta-analysis adhered to the Preferred Reporting Items for Systematic Review and Meta-analyses 2020 guidelines.

### Search Strategy

A comprehensive literature search was done using MEDLINE, EMBASE, PubMed, and Web of Science. The keywords “single stage revision,” “exchange arthroplasty” “periprosthetic infection,” “PJI,” and “single stage” were implemented in the search. Only English language studies were included. All studies published through July 2020 were considered. Institutional review board approval was not obtained because the study did not require direct contact with patients or patient identifying medical record review.

### Inclusion and Exclusion Criteria

Two authors independently reviewed the eligibility of articles in the study. Studies needed to include a series of patients evaluated prospectively or retrospectively undergoing single-stage revision arthroplasty for PJI with minimum 1-year follow-up data. Baseline demographic data, implant survivorship, and causes of revision surgery needed to be documented during the specified follow-up time. Review papers, editorials, and commentaries were excluded.

### Data Extraction

Relevant data extracted from each study included primary author, year of publication, level of evidence of study, number of months of follow-up, number of patients, and demographic information, including average age, sex, average body mass index, and number and cause of revision surgeries in each group.

### Primary and Secondary Outcomes

The primary outcomes of the study were rates of revision surgery secondary to infection compared with aseptic loosening for all procedures and THA and TKA specifically. Secondary outcomes included the overall revision surgery rate, revision surgery rates from other etiologies, and mortality rate.

### Statistical Analysis

Raw data from the included studies in the meta-analysis were converted to weighted averages based on the number of subjects in each group for age and follow-up time. A pooled frequency for sex was calculated as well. The frequency of all-cause revision surgeries and revision surgeries secondary to specific causes, and mortality rate were determined through a binary random-effects meta-analysis using the DerSimonian-Laird method. This was done for all single-stage revisions and THA and TKA procedures separately. The difference in revision surgery frequency for infection and aseptic loosening specifically was calculated with the same method, and a 95% confidence interval (CI) was constructed to determine statistical significance.

## Results

### Study Selection

**Figure 1:**
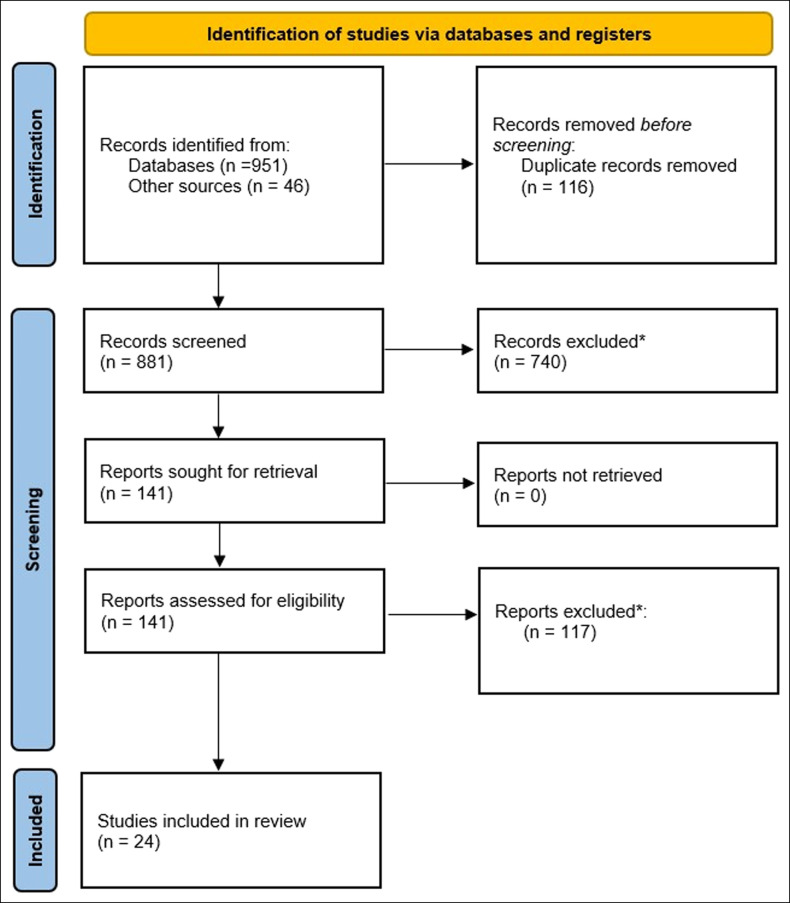
This flowchart depicts the Preferred Reporting Items for Systematic Review and Meta-analyses 2020 flow diagram for selection of studies in this meta-analysis. *Reasons for exclusion: case reports, wrong procedures (two-stage revision, arthrodesis, salvage procedures, metatarsal implants, and spine procedures), unclear if patients underwent single-stage or two-stage revision, animal studies, patients younger than 18 years, and inadequate/absent data on aseptic loosening.

A total of 997 potential articles were identified through keyword search in the electronic databases and other sources. After removal of 116 duplicates, 881 records remained for screening. Seven hundred forty articles were removed because of incorrect surgical procedure, use of animal studies, presence of case reports, and absent aseptic loosening data, yielding 141 articles for additional review. Twenty-four articles remained in the meta-analysis after applying exclusion criteria. Details of the screening process are presented in the Preferred Reporting Items for Systematic Review and Meta-analyses flowchart in Figure 1.

### Included Studies and Baseline Characteristics

The 24 included studies were published between 1984 and 2020.^4,7,8,9,10,11,12,13,14,15,16,17,18,19,20,21,22,23,24,25,26,27,28,29^ The total number of revision surgeries was 2,209, with single-stage revision TKA comprising 5 studies and 147 revision surgeries and single-stage revision THA comprising 19 studies and 2,062 revision surgeries. Table 1 presents all included studies with level of evidence and type of surgery as well as baseline characteristics of percentage of men, mean follow-up time, mean body mass index, and mean age, where data were present. Table 2 summarizes the total number of revision surgeries and reasons for revision for each study.

**Table 1:** Included Articles in Meta-analysis With Demographic and Study Data

Study Primary Author	Publication Year	Level of Evidence	Men (%)	Follow-up^a^ (mo)	BMI^a^ (kg/m^2^)	Age^a^ (yr)
Buchholz	1984	3	—^b^	52	—	—
Zahar	2016	4	66	120	—	70
Wroblewski	1986	2	53	38	—	63
Zeller	2014	2	58	41.6	27	71
Callaghan	1999	3	50	109	—	65.3
Klouche	2012	3	53	35	28	63.6
Tibrewal	2014	2	34	126	—	66.8
Haddad	2015	3	50	78	—	63
Ji	2019	3	54	58	25.7	58.7
Loty	1992	3	—	47.3	—	65.7
Svensson	2019	2	61	131	—	70
De Man	2011	3	45	40	—	67
Wilson	1989	2	57	41	—	51.3
Bori	2014	3	38	45	—	72.4
Ilchmann	2016	3	51	79	—	—
Labruyère	2015	4	—	60	25.5	67
Klatte	2014	3	44	36	—	71.4
Wang	2020	4	—	35.67	24.3	—
Yoo	2009	3	67	86	—	50
Raut	1996	2	60	96	—	65.1
Chalmers	2020	2	—	36	29	66
Rahman	2017	3	40	103	30	58.93
Winkler	2012	3	—	52.8	—	—
Hongbin	2014	3	58	41	—	46.3

BMI = body mass index

a Mean values are provided.

b No data available.

**Table 2:** Revision Procedures and Number of Revision Surgeries by Etiology

Study Primary Author	No. of Single-stage Revisions	Revision Procedure	All Causes^a^	Infection^a^	Aseptic Loosening^a^	Instability^a^	Fracture^a^	Mortality^a^
Buchholz	825	THA	182	147	35	—	—	—
Zahar	59	TKA	12	5	7	70	1	2
Wroblewski	102	THA	2	2	0	63	0	7
Zeller	157	THA	15	6	9	71	—	2
Callaghan	24	THA	10	2	1	65.3	—	12
Klouche	38	THA	1	0	0	63.6	0	0
Tibrewal	50	TKA	10	1	9	66.8	—	0
Haddad	28	TKA	0	0	0	63	0	0
Ji	111	THA	12	7	0	58.7	5	3
Loty	90	THA	15	8	7	65.7	—	4
Svensson	404	THA	83	28	35	70	—	—
De Man	22	THA	1	0	2	67	4	0
Wilson	7	THA	1	0	1	51.3	0	0
Bori	24	THA	1	1	0	72.4	0	—
Ilchmann	39	THA	4	0	4	—	0	0
Labruyère	9	TKA	0	0	0	67	0	0
Klatte	50	THA	8	2	0	71.4	0	0
Wang	24	THA	0	0	0	—	0	0
Yoo	12	THA	1	0	1	50	0	0
Raut	15	THA	2	1	1	65.1	0	0
Chalmers	1	TKA	0	0	0	66	0	0
Rahman	15	THA	3	1	1	58.93	0	0
Winkler	91	THA	8	8	0	—	0	0
Hongbin	12	THA	0	0	0	46.3	0	0

THA = total hip arthroplasty, TKA = total knee arthroplasty

a Etiology and number of revision surgeries.

### Primary and Secondary Outcomes

**Figure 2:**
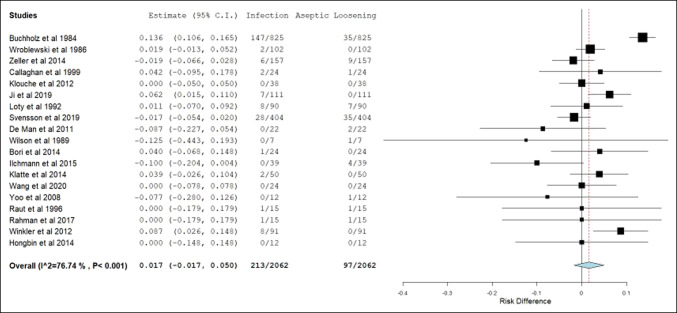
This forest plot illustrates the risk difference between infection and aseptic loosening as etiologies of revision surgery and the associated 95% CI after single-stage revision total hip arthroplasty. *CI = confidence interval

**Figure 3:**
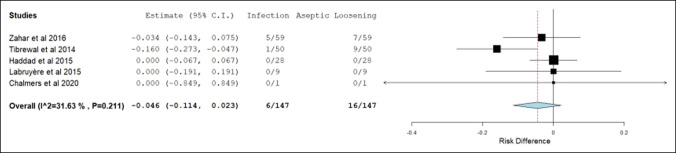
This forest plot illustrates the risk difference between infection and aseptic loosening as etiologies of revision surgery and the associated 95% CI after single-stage revision total knee arthroplasty. *CI = confidence interval

Table 3 presents the pooled and meta-analyzed data organized by revision THA and TKA. Weighted mean follow-up, weighted mean age, and meta-analyzed frequencies of revision surgeries by etiology are presented. The overall all-cause revision surgery rate was 11.1% for single-stage revision THA and 11.8% for single-stage revision TKA. The frequency of revision surgery secondary to infection and aseptic loosening for THA was 5.5% and 3.3%, respectively. The frequency of revision surgery secondary to infection and aseptic loosening for TKA was 3% and 8.8%, respectively. Figures 2,3 illustrate forest plots of septic and aseptic loosening risk differences and the respective 95% CIs for THA (1.7%, [−1.7% to 5.0%]) and TKA (−4.6%, [−11.4% to 2.3%]). As the 95% CI contains 0, no statistical difference between the frequency of revision surgery due to infection and aseptic loosening existed. Revision surgeries due to instability and fracture combined were less than 3% and mortality 2.5% and 1.7% in the THA and TKA groups, respectively.

**Table 3:** Pooled Data and Meta-analysis of Revision Surgery Frequency

Revision Procedure Type	No. of Procedures	Weighted Follow-up^a^ (mo)	Weighted Age^a^ (yr)	Men^b^ (%)	All Cause^c^ (%)	Infection^c^ (%)	Aseptic Loosening^c^ (%)	Instability and Fracture^c^ (%)	Mortality (%)
THA only	2,062	67	66.2	55.7	11.1	5.5	3.3	2.7	2.5
TKA only	147	110	67.4	51.1	11.8	3	8.8	1.9	1.7

THA = total hip arthroplasty, TKA = total knee arthroplasty

a Mean values are provided.

b Pooled frequency of men across studies.

c Etiology and frequency of revision surgeries.

## Discussion

This study sought to determine rates of etiologies of revision surgery after single-stage revision arthroplasty for PJI. Twenty-four studies of single-stage revision TKA and THA were included in the meta-analysis after a methodical screening process from electronic databases. The overall all-cause revision surgery rate was 11.1% for single-stage revision THA and 11.8% for single-stage revision TKA. The revision surgery rate attributable to infection was not statistically different from the rate attributable to aseptic loosening in both single-stage revision THA and TKA. The revision surgery rate due to instability and fracture combined and mortality rate were both <3% in each group at greater than 5 years of weighted follow-up.

The revision surgery rate secondary to infection was low in this meta-analysis. This study estimated a failure rate of 5.3% and 3.0% due to infection after single-stage revision THA and TKA, respectively, illustrating the efficacy of this surgical management strategy for PJI. Infection-free success after single-stage revision arthroplasty is variably cited between 77% and 100% in the literature (20, 32). This large range is likely attributable to variations in surgical technique, patient selection and associated host comorbidities, chronicity and severity of infection, and other factors between studies.^30^ The success of single-stage revision arthroplasty is maximized when three principles are adhered to identification of infecting organism with sensitivities and minimum inhibitory concentrations known before surgery, radical débridement of infected tissues, and delivery of local and systemic antimicrobial therapy.^4^ This meta-analysis helps verify that single-stage revision is an acceptable alternative to two-stage revision in the right patient population.

At a weighted follow-up of nearly 10 years in this study, the failure rate secondary to aseptic loosening in single-stage revision TKA was noted to be approximately 8.8%. By contrast, at 15 years of follow-up, the overall failure rate for primary TKA is only 5% to 10%, with aseptic loosening comprising 20% to 30% of failures.^31,32,33,34,35,36,37^ This discrepancy is multifactorial but could be related to débridement strategy and implant selection. A key principle of single-stage revision is radical resection of infected tissue unlike intralesional débridement in a two-stage revision. In PJI of the knee, en bloc resection of the synovial membrane, infected soft tissue and bone, and sometimes collateral ligaments are required.^3^ Constrained, hinged, and/or stemmed implants are often required, which can increase shear forces on the polyethylene bearing and hasten the osteolysis process from debris generation. Another reason could be that the extensive bone débridement reduces the surface area for bone cement interdigitation. As cemented implants are often used in revision surgery for PJI, cement fixation may be suboptimal and reduce prosthesis survival.

Strengths of this study include high statistical power with a total of 2,062 single-stage revision THA procedures analyzed, and a weighted overall follow-up of greater than 5 years. Although the single-stage TKA number was lower at 147, the weighted follow-up was greater than 9 years. Limitations include the use of mostly retrospective or case series studies in the meta-analysis and heterogeneity of studies. Furthermore, although the single-stage THA number was high, about half of these procedures were done before 2000. This limits external validity because older surgical techniques and technology may have led to different complications and failure mechanisms. Finally, the higher aseptic loosening rate of single-stage revision TKA noted in this study was compared with existing data from the literature, precluding a statistical comparison to determine the significance of this difference.

Future studies should focus on the use of noncemented components in revision arthroplasty for PJI. Noncemented components can address drawbacks of poor cement fixation in the setting of bone loss with infection. Edwards et al^38^ demonstrated that the use of a diaphyseal-engaging noncemented stem in two-stage revision TKA had markedly lower radiographic evidence of loosening compared with cemented stems. Despite antibiotic cement spacers not being used for local antibiotic delivery, infection-free survival after noncemented revision for PJI still seem quite high. In two studies of noncemented THA in revision PJI using intra-articular infusion or topical powder for local antibiotic delivery, infection-free survivorship ranged from 90% to 95% at 4 to 5 years of follow-up.^13,29^

This meta-analysis of single-stage revision THA and TKA for PJI demonstrated overall high rates of survivorship, low mortality, and revision surgeries secondary to infection and aseptic loosening. The aseptic loosening revision surgery rate of single-stage revision TKA was found to be higher than that of primary TKA as reported in the literature. Single-stage revision arthroplasty for PJI may offer a reliable treatment strategy in a selected patient population. Surgeons should consider aseptic loosening as an equally common complication as infection in single-stage revision arthroplasty.
